# Kind Words from Friends, with Comments

**Published:** 1872-08

**Authors:** 


### KIND WORDS FROM FRIENDS, WITH COMMENTS.

"What is the matter with Dr. Gaillard? He must be bewildered. If he thought the editors of the COMPANION did wrong, or violated ethics, in publishing complimentary letters concerning their journal, why not discuss the points at issue? If he believed, from the evidence, that Dr. P. and the entire profession of Georgia (save about forty), had done wrong, legally, morally and professionally, in opposing the errors and irregularities of the Atlanta Medical College and its Faculty, why not discuss the subject or subjects in a manly and dignified manner—that the readers of both journals might be benefitted? It is very clear, to my mind, that Dr. G.'s motives are not very commendable, or that he feels he is badly whipped in the controversy, and has not the moral courage to acknowledge it. If he has read the record, (and he ought to have done this before attempting his review), he is compelled to know that Dr. Powell was never expelled, or could not be by any action of the Faculty, and that he has never given an opinion or made a statement that has not been sustained by the Legislature of Georgia, the Trustees of the Atlanta Medical College, the Georgia Medical Association, and the Fulton County Medical Society. This record would also teach him that every statement made by this Faculty, in print, at least, has been declared, either by the Trustees, the Georgia Medical Association, or the Fulton County Medical Society, to be false. But I repeat my former advice, viz: to waste but little time on the charge that you were expelled, and its causes. The profession generally are satisfied on all these issues."

Our friend states his points clearly. The querulous editor of

Louisville has our sympathies. We always feel deeply for any man who, under whatever influence, chooses rather to caress his spleen than to frankly and manfully own a fault.

Our correspondent has, probably, given the only true solution of the difficulty besetting the Louisville critic. He thinks he is probably "bewildered." We would suggest, however, that he may be be-witched; and, in all kindness, paraphrasing the words of inspired truth, we would mournfully inquire—"O! foolish" editor, what "hath be-witched you?" And the only response likely to satisfy the query is—*Hasheesh!* The strange and wonderful properties of *hasheesh* are well known. Hallucinations, with their self-glorying fancies, weaving gorgeous visions through the brain, deluding the hasheesh-eater, under the influence of this wonderful drug, are truly marvellous. A small bolus is swallowed—and what enrapturing visions marshal themselves into view! The duped soul revels in the strangest delusions of self-exaltation. He dreams of self, solely—wields a sceptre of imaginary authority, speaking great words swollen with the weight of infallibility. He essays to "out Herod, Herod," in an innumerable role of conflicting characters. At one time he is the spirit and embodiment of a Chesterfield, strutting in self-robed gewgaws of imaginary "decorum and propriety," discussing their rules, and outraging them in every act. He perches himself upon the seat of the law-giver, mourning over violated ethics, and dashes them petulantly to the ground when they come trouping in his way. He soars a very eagle in the upper ether of his frenzied imagination —fuss, feathers and all—and sadly laments the want of appreciation of his friends in their failure to behold in him the likeness of a "bug" or a "snake"—conditions suggestive of creeping and crawling, which some less conscientious mortals are wont to do, over the good names and reputation of their brethren. Woful, woful indeed, is the *hasheesh-eater* in the hasheesh state!

We can excuse the hasheesh-eater, erratic as he is, cutting such

"Fantastic tricks before high heaven
As make the angels weep!"

Like the poor Indian, who sought to ascribe his actswhen drunk, not to reason, but to whisky, so we soothingly pat the hallucinated head of our unfortunate friend, and sob out—*hasheesh!* It cuts the soul to the quick to see a friend in such a plight.

We pity the hasheesh-eater. Who would not? Hasheesh takes a man out of himself. It metamorphoses a good man, whose impulses are noble and true, into the most wretched and querulous of mortals. It coagulates the milk of human kindness in a breast otherwise warm with generous instincts, and transmutes all generosity into vinegar and gall. It sours the mind with its acid phlegm, and begets the most loathsome and distressing mental and moral dyspepsia. The victim growls and snaps upon all men and events. It does this and more. O! how the "the world, the flesh and the devil" ought to pity the hasheesh-eater!

Yet strange—very strange—the hasheesh-eater constantly torments his soul in solving one question. It looms before him ever. It will not down at his bidding. It distresses him beyond endurance. He cannot see, for the life of him, why all men do not fall down and worship him! Poor hasheesh-eater!

In the hasheesh state everything is a failure. Every medical man and journal in the land is a failure—every college is a failure—every editor is a failure—all, save one. The late President of the American Medical Association was a failure—the hasheesh-eater would not have been. The Association of editors was a failure—he was not one of them. The National Association was a huge failure—he could have made it a success if he had only gone. Medicine weeps that he did not! O! cruel hasheesh-eater, why did you "stay away!"

Yet, amidst all this woe, one mercy is left the hasheesh-eater: He occasionally has a lucid interval. If the good genius of our poor brother—dearly beloved and pitied—should vouchsafe to bestow upon him so great a blessing "just now," we would tenderly and affectionately whisper a word in his ear.

Dear friend and brother, we condole with you. Here, take our hand, as we advise you—believe us, it is for your good. Now, brother, as we look you in the eye, we implore you, as you love your soul, to stop hasheesh. It is murdering your prospects. It threatens to make—yea, has already made—your name the synonym of growler, wrangler, and capricious meddler. It threatens to drive away many warm-hearted friends. It has made "the largest medical monthly in America" the most inglorious of "*pepper-boxes*,"—through which friends have been peppered; colleges have been peppered; societies have been peppered; associations have been peppered—in fact, everything peppered, but the one idolized chief of the "*Pepper-Box.*" O! the misery of hasheesh folly! Why, O, why, infatuated editor, will you so use your "Pepper-Box?" Why not let it sprinkle in its legitimate culinary way, undistressed by hasheesh absurdities? It is, and has been a good "Pepper-Box," in many respects—in fact in nearly all respects, save only, when hasheesh abounded. Truly you know Yandell would not have been disturbed, and Mobile would have gone on in peace—*from you*—in view of your local surroundings, and that huge beneficiary list, with its "Hippocratic Oath" and all—had not hasheesh prevailed. So, we beg you to stop it. It will ruin you. It will dry up the fountain of "the largest medical monthly in America." It will seal up the doors ef the medical institution of which you are the mouth-piece—in spite of its able faculty and good position—peppered to death by the universal,. all-scattering "Pepper-Box" in your hasheesh hands—riddled, lacerated, mangled, under the weight of that beneficiary list—the mighty swing of that "Hippocratic Oath," and the wailings and laments of the "Pepper-Box," in notes so discordant to its practice on "cheap schools" and "low fees!"—Hold up—we beg you! If not able—if hasheesh moves you beyond your power—beg your colleagues to arrest the Sampson-like efforts you are making for the destruction of your common temple! If they cannot—if the fatal habit has "grown with your growth and strengthened with your strength" to such an extent as to render their efforts powerless—then we pity you and them. In soberness and truth we say—brother dear and tender—they had better start you on your rounds again. From Richmond you came—from Louisville you may have to go. Like the Wandering Jew—on and on—you must tramp from city to city, from college to college (?)—"Pepper-Box" and all. In such a case, none could or would deplore your pitiable end more than your friends of the COMPANION—Associates and all—an end whose only glory would be the long list of cities which had covered the title-page of "the largest medical monthly in America."

"I, too, thank the editors of the COMPANION for all they have said on ethics, and particularly thank the brother who suggested in the July number, "that the profession make a compact" for the elevation of medical education. His plan is the only one that will succeed, and I do hope every honorable physician will adopt it at once. It is the just plan—it is in accordance with our ethics, and unless it can be adopted, we had better abolish the ethics. It will not be denied that inferior schools are flooding the country with quacks and unqualified physicians, and that in many localities old and scientific men are being starved and driven out of the profession by these imposters. As our brother says, the ethics forbid our consulting with these characters; and why should it allow first-class colleges to recognize these little one-horse colleges? They have no right to recognize them. It is a violation of the ethics. It is unjust to themselves, to their educated brethren, and to the afflicted. There are a few colleges that have taken this high stand. I hope preceptors everywhere will advise, and see to it, that their students attend them. It is the only plan that will enoble the profession, advance the science, and protect its members and the sick. The COMPANION is the only independent journal published in the South. The profession will expect it to speak out. Don't be afraid; the good men everywhere will sustain you. It is essential, to sustain the interest and character of the profession South, that they have an independent journal. For the honor of the profession North, East and West, such an one must and will be sustained. So far the COMPANION has acted nobly in adhering to principle, and upholding the dignity and honor of the profession in every section—showing no prejudice or partiality—and we unite with our unknown brother, in his request to repeat its efforts, and "call upon the profession in thunder-tones never to allow any young man to become a student in their offices unless well and fully qualified by education and morals. And never—no, never—allow any student to take even his first course of instruction in any but the best and most thorough colleges in the country."

The above needs no comment. We have requested our friends to send us paragraphs of such things as they are interested in, and we still invite them to do so. We are the friends of every institution North, South, East or West, that deserves the confinence of the profession, and will take pleasure in saying something good about them, as opportunity may present. Our advertising columns shall be open to all such; and we may (as a matter of duty as journalists), notice others, to show their want of merit, and claims to the confidence of the profession.

